# Assessment of Mitochondrial DNA Copy Number in Progressive Supranuclear Palsy Patients: Evidence From a Pilot Study

**DOI:** 10.1111/ene.70594

**Published:** 2026-04-17

**Authors:** Luigi Citrigno, Annamaria Cerantonio, Monica Gagliardi, Radha Procopio, Alessia Felicetti, Anna Aureli, Maurizio Morelli, Grazia Annesi

**Affiliations:** ^1^ Institute for Biomedical Research and Innovation, National Research Council Cosenza Italy; ^2^ Department of Medical and Surgical Sciences, Neuroscience Research Center Magna Graecia University Catanzaro Italy; ^3^ Department of Medical and Surgical Sciences Institute of Neurology, Magna Graecia University Catanzaro Italy; ^4^ Institute of Translational Pharmacology, National Research Council L'Aquila Italy

**Keywords:** biomarker, mitochondrial dysfunction, mtDNA copy number, progressive supranuclear palsy, tauopathy

## Abstract

**Background:**

Progressive supranuclear palsy (PSP) is a rare neurodegenerative tauopathy characterized by early postural instability and vertical gaze palsy. Mitochondrial dysfunctions have been increasingly implicated in the pathogenesis of neurodegenerative disorders, including PSP. We investigated mitochondrial DNA copy number (mtDNA‐CN) alterations in the peripheral blood of PSP patients, assessing its potential as a biomarker for disease onset and progression.

**Methods:**

We measured mtDNA‐CN in a cohort of clinically diagnosed PSP patients and age‐matched healthy controls using quantitative real‐time PCR. We evaluated differences across clinical phenotypes and age groups.

**Results:**

PSP patients exhibited a significant reduction in ND3‐CN compared to healthy controls (*p* < 0.0001). This depletion remained consistent across age groups, suggesting that mitochondrial impairment in PSP is independent of physiological aging. Although not statistically significant, ND3‐CN levels were lower in PSP‐parkinsonism (PSP‐P) patients compared to those with Richardson's syndrome (PSP‐RS). Interestingly, in PSP‐RS patients, ND3‐CN levels tended to increase with age, potentially reflecting an age‐related compensatory mitochondrial response to chronic neuroinflammation.

**Conclusions:**

Our findings support the involvement of mitochondrial dysfunction in PSP pathogenesis, suggesting that peripheral mtDNA‐CN may serve as a non‐invasive biomarker for disease monitoring. Further studies in larger cohorts are warranted to validate its prognostic potential in different PSP phenotypes.

## 1 Introduction

Progressive supranuclear palsy (PSP) (MIM #601104) is a rare, sporadic neurodegenerative parkinsonian syndrome with a reported prevalence ranging from 1 to 18 individuals per 100,000 [1]. The classical presentation of PSP is characterized by tauopathy and a symmetrical akinetic‐rigid syndrome (atypical parkinsonism), along with vertical supranuclear gaze palsy. Patients often exhibit gait instability, frequent backward falls, and early cognitive and behavioral changes [2].

PSP occurs more frequently in males and typically follows a rapidly progression, with a mean age of onset around 63 years and a median survival of 6.9 years [3].

Diagnostic criteria for PSP, developed by the Movement Disorder Society, encompass clinical features, neuropathological findings, therapeutic responses, and neuroimaging evidence [4].

PSP includes several phenotypic variants, providing prognostic insights. PSP‐Richardson syndrome (PSP‐RS) is the most common subtype, accounting for 76% of autopsy‐confirmed cases and is associated with a poorer prognosis [5].

The PSP‐parkinsonism (PSP‐P) variant, the second most frequent subtype, shares several clinical features with idiopathic Parkinson's disease (PD), including asymmetrical tremor, non‐axial dystonia, and early bradykinesia, along with a significant response to levodopa. PSP‐P patients typically have a longer survival time (9.1 years) compared to those with PSP‐RS. Nonetheless, differential diagnosis remains challenging due to overlapping clinical characteristics [6].

Recently the inflammatory hypothesis has been widely investigated to deeper explain the process of neurodegeneration underlying this disease and specific neuroinflammatory profiles have been established to highlight differences in the pathomechanism responsible for the distinct PSP clinical phenotypes [7]. For example, different concentrations of interleukin‐1 beta (IL‐1β) and IL‐6 have been observed in serum and cerebrospinal fluid (CSF) among PSP‐P and PSP‐RS patients, and the lowest concentration of these interleukins was detected in the latter group, where a more rapid deterioration and a more severe atrophy in subcortical structures take place [8]. In another work, hepcidin, a peptide which controls the distribution of iron in tissues, has been evaluated to circumvent the overlap between neurodegeneration and inflammatory mechanisms in the pathogenesis of PSP. Authors detected a significant increase of serum hepcidin only in PSP‐RS patients, suggesting that the increase of this peptide could be a feature linked with the more pronounced severity of motor symptoms and neurodegeneration which mainly affects this phenotype [9]. Moreover, it has been hypothesized that the expression of Glial Cell Line‐Derived Neurotrophic Factor (GDNF) could have a possible impact on the different clinical course of these two PSP phenotypes. To this end, Alster et al. detected an increase of GDNF levels in the CSF of PSP‐RS patients and a concomitant higher concentration of this protein in the serum of PSP‐P patients, indicating that PSP‐RS patients might be affected by accelerated transition from “peripheral” to “central” GDNF activation [10]. These evidences demonstrated that in PSP there might be a specific neuroinflammatory pattern indicating differences in inflammatory activity which could mirror the differences in PSP phenotypes and their clinical course [11].

Neuropathologically, PSP is marked by primary four‐repeat (4R) tauopathies with neurofibrillary tangles (NFTs), tufted astrocytes (TAs), astrocytic plaques (APs), neuropil threads (NTs), and oligodendroglial coiled bodies (CBs), predominantly in the basal ganglia, diencephalon, and brainstem [12].

Although PSP has traditionally been considered a sporadic and age‐related disorder, growing evidence supports a genetic component. The *MAPT* gene, encoding the microtubule‐associated protein tau, is a well‐established genetic risk factor [13].

Specifically, the H1/H2 haplotype at chromosome 17q21.31, a common inversion polymorphism in *MAPT*, is associated with increased PSP risk. Other genome‐wide significant variants (GWS; *p* < 5 × 10^−8^) have been identified near *STX6*, *EIF2AK3*, and *MOBP*. Additionally, the rs2242367 variant within an intron of the *SLC2A13* gene, located near the PD/parkinsonism‐associated gene *LRRK2*, may influence PSP disease duration [14]. Other loci adjacent to *RUNX2*, *SLCO1A2*, and *KANSL1* have shown potential involvement in PSP heritability, although the exact mechanisms through which genetic factors influence disease pathogenesis remain unclear [15].

To date, only a limited number of biomarkers have been proposed to improve PSP diagnosis. Among them, cerebrospinal fluid (CSF) levels of total tau (t‐tau), phosphorylated tau (p‐tau), and neurofilament light chain (NfL) have been investigated as indicators of disease progression and axonal degeneration [16].

Plasma NfL levels have also been correlated with worse motor and cognitive performance in PSP patients, supporting their role in disease severity assessment [17].

Recently, microRNAs (miRNAs) have emerged as potential biomarkers due to their ability to suppress target gene expression and modulate neurotoxic protein activity, thereby influencing tau isoform regulation [18]. However, their clinical utility is limited by heterogeneous study results and a lack of robust experimental validation [18].

In addition, PSP has been linked to mitochondrial energy production deficits, as evidenced by decreased glucose metabolism and elevated oxidative stress in post‐mortem brain tissue [19].

Emerging studies have highlighted the role of mitochondrial dysfunction in neuroinflammation, a process termed “mitoinflammation” [20]. Cellular stress may trigger mitochondrial DNA (mtDNA) damage and fragmentation, activating inflammatory pathways such as the NOD‐Like Receptor Protein 3 (NLRP3) inflammasome and the cyclic GMP‐AMP Synthase–Stimulator of Interferon Genes (cGAS–STING) pathway. In this context, mtDNA can act as a mitochondrial damage‐associated molecular pattern (mtDAMP) recognized by microglial immune receptors, thereby exacerbating neuroinflammation [20].

“Mitoinflammation” is also associated with a reduction in mitochondrial DNA copy number (mtDNA‐CN), implying that alterations in mitochondrial content may contribute to PSP pathogenesis [20].

To date, no studies have assessed blood mtDNA‐CN levels in PSP patients to determine their potential role in disease risk or severity.

In this preliminary study, we evaluated mtDNA‐CN levels in a cohort of PSP patients from Southern Italy, aiming to explore the potential role of mtDNA alterations in the pathogenesis of PSP.

## 2 Materials and Methods

### 2.1 Selection of Subjects

A total of 50 pathologically confirmed PSP cases and 64 neurologically healthy controls, matched for sex and age, were selected for this study. All participants, of Caucasian origin, were born in Calabria (Southern Italy) and were recruited at the Neuroscience Research Center, Magna Graecia University of Catanzaro, Italy.

The PSP cohort was examined by neurologists specialized in movement disorders and diagnoses were made according to the criteria established by The Movement Disorder Society [4].

All participants signed informed consent. Peripheral blood samples were collected and further used for total DNA extraction.

We decided to use peripheral blood to study mitochondrial dysfunctions and content because of the ease of collection, processing and storage of this biologic fluid. In our opinion this choice represents a very cost‐effective option which does not require further invasive methods (as compared with biopsies or CSF fluid collections). Moreover, blood cells are able to circulate through the entire body, thus reflecting all the changes in mitochondrial activity (inflammation, oxidative stress) that could influence the systemic condition of individuals.

### 2.2 DNA Extraction and mtDNA Copy Number Evaluation

Total DNA was extracted from blood samples using standard methods. DNA concentrations were determined using Qubit dsDNA BR (Broad Range) Assay Kit on Qubit 3.0 Fluorometer (Invitrogen).

All the experiments for mtDNA‐CN evaluation have been performed at the Institute for Biomedical Research and Innovation (IRIB‐CNR) of Mangone (CS), Italy.

Quantitative Real‐Time PCR (qPCR) was carried out to quantify mtDNA‐CN levels in DNA samples of PSP patients and healthy controls using two specific primer sets for the amplification of *ND3* mitochondrial gene and for tyrosine hydroxylase (*TH*) nuclear gene.

Samples were run in triplicate in 384‐well reaction plates on ABI PRISM 7900 Real‐Time PCR device (Applied BioSystems). In each well, qPCR was performed in 20 μL of final reaction volume containing 10 μL of SYBR Master Mix, 2 μL of both forward and reverse primers, 1 μL (5 ng) of DNA, and 5 μL of sterile purified water.

A standard curve using serial dilutions of a reference DNA was included to determine the amplification efficiency of both *ND3* and *TH* primers.

Cycler conditions were: one cycle of 10 min at 95°C; 40 cycles of 15 s at 95°C, 20 s at 53°C and 45 s at 72°C.

### 2.3 Statistical Analysis

The N/S ratio (‐dCt) for each sample was calculated by subtracting the average of *TH* Ct value from the average of ND3 Ct value. Standard curve points included in each 384‐well plate were used as calibrator DNA to reduce inter‐assay variability. The relative N/S ratio (‐ddCt) was calculated by subtracting the N/S ratio of the calibrator DNA from the N/S ratio of each sample.

Statistical analyses were performed using Graph Pad Prism (Graph Pad Software Inc., San Diego, CA, USA) version 10.4. The Student's *t*‐test was used to evaluate differences in normally distributed data, while the Mann Whitney test was applied to compare differences in non‐normally distributed data.

Multiple linear regression and Pearson correlation coefficient were employed to analyze associations between mtDNA‐CN and confounding factors, such as age, in both PSP patients and controls.

Statistical significance of differences between and within groups was determined by mean values of two‐way ANOVA, followed by Šidák and Tukey's multiple comparisons tests.

All results were considered significant when *p* < 0.05 and no correction for multiple testing was applied in this exploratory study.

## 3 Results

### 3.1 Demographic and Clinical Characteristics of Participants

Clinical and demographic characteristics of PSP patients and healthy control (CTR) individuals have been summarized in Table 1.

**TABLE 1:** Demographic and clinical characteristics of subjects recruited for the study.

	CTR	PSP
𝑛 (M; F)	64 (37; 27)	50 (34; 16)
Mean age ± SD (years)	70.5 ± 6.2	70.5 ± 7.1
Clinical phenotype (*n*)	—	PSP‐P (18)
	—	PSP‐RS (33)
	—	PSP‐PGF (1)

The PSP cohort included 50 patients (34 males and 16 females), with a mean age of 70.5 ± 7.1 years.

Based on clinical phenotype, PSP patients were further clustered in 17 PSP‐P and 33 PSP‐RS patients.

A control group without neurological disorders consisting of 64 healthy subjects (37 males and 27 females) with a mean age of 70.5 ± 6.2 years was also examined.

### 3.2 mtDNA CN Levels in PSP Patients

**FIGURE 1:**
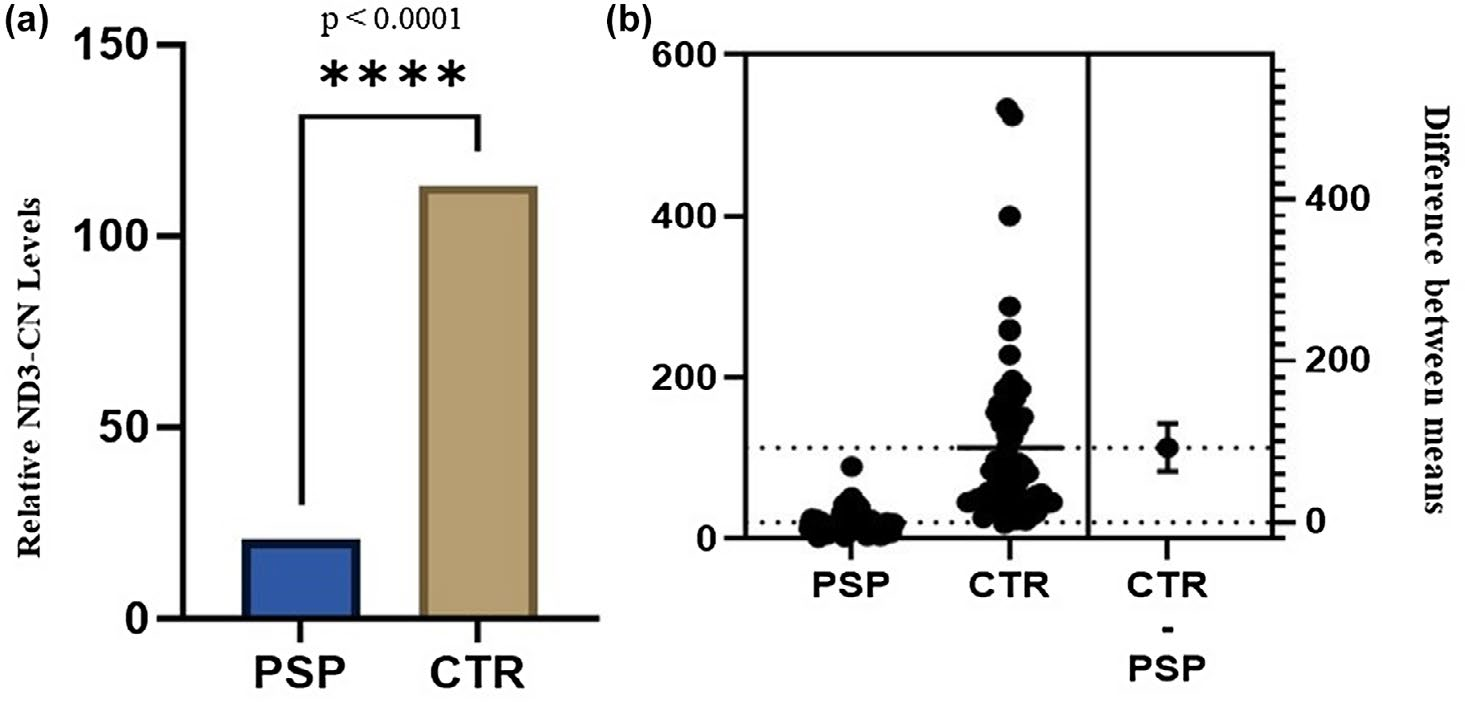
(a) Relative ND3 copy number (CN) content in progressive supranuclear palsy (PSP) patients compared to healthy controls. (b) Difference between mean among PSP patients compared to healthy controls.

Since mtDNA‐CN levels were shown to decline in the majority of neurodegenerative disorders, we investigated whether a similar reduction occurs in PSP. A strong significant reduction of ND3‐CN levels was observed in PSP patients compared to CTR group (mean ± SD; PSP 20.7 ± 15.6 versus CTR 113.3 ± 106.4; Mann–Whitney *p* < 0.0001; Figure 1).

Moreover, we investigated if these changes were also noticeable in both groups after clustering them for gender and age.

**FIGURE 2:**
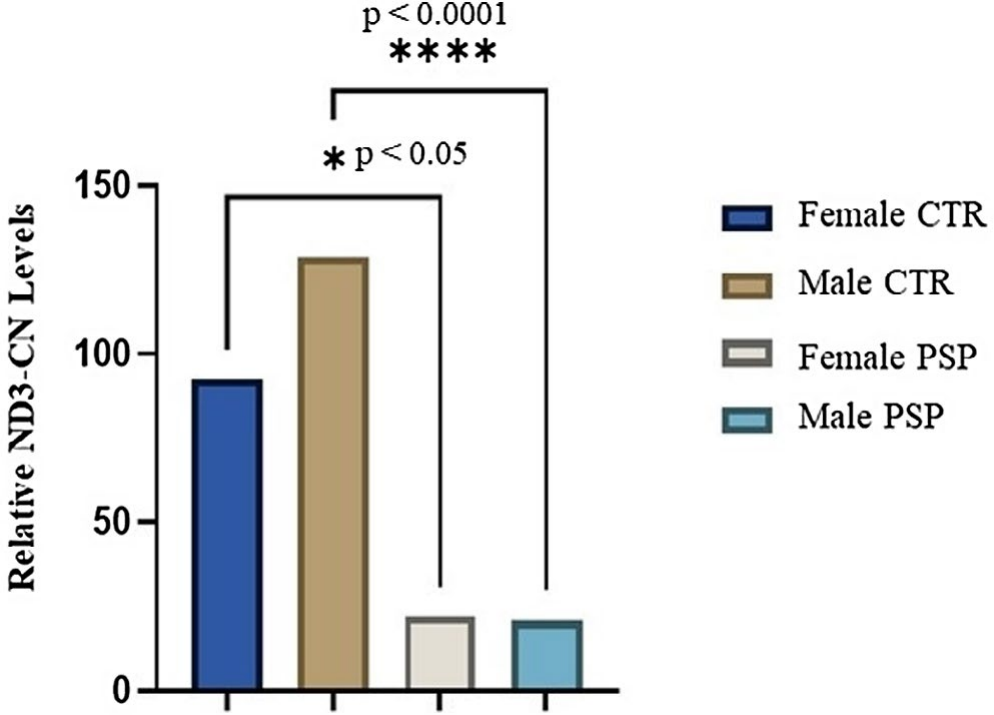
Relative ND3 copy number (CN) levels measured in progressive supranuclear palsy (PSP) patients compared to healthy controls clustered for sex.

Two‐ way Anova analysis revealed a significant decrease in ND3‐CN content in PSP patients compared to CTR clustered for gender, and the highest score was obtained when comparison was performed between males PSP patients and males of CTR group (*p* = 0.0001) (Figure 2).

A comparable result was found when we analyzed the differences between mtDNA‐CN levels and age in PSP patients and CTR subjects stratified in four age groups (group 1: 58–65 years; group 2: 66–70 years; group 3: 71–75 years; group four: 76–85 years).

**FIGURE 3:**
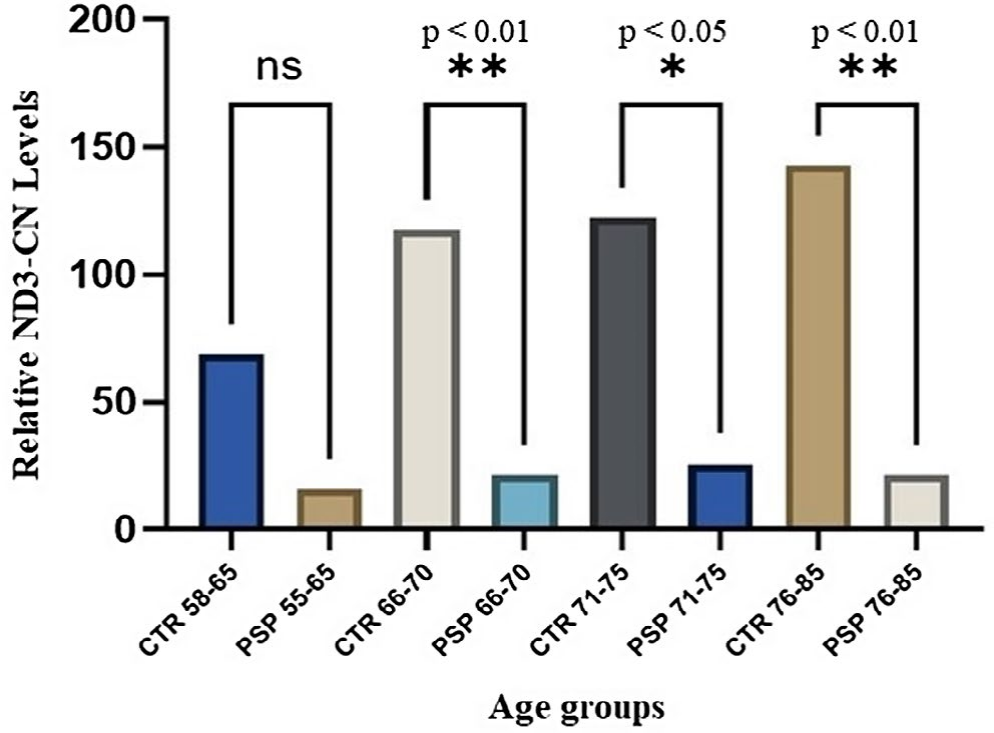
Relative ND3 copy number (CN) levels measured in progressive supranuclear palsy (PSP) patients compared to healthy controls clustered for age.

As reported in Figure 3, apart from group 1, a tendency of significantly lower ND3‐CN levels, even in the elderly age groups, was observed in all PSP patients compared to CTR groups.

We also investigated if changes in mtDNA‐CN content were a parameter able to discriminate PSP patients on the basis of their clinical phenotypes.

**FIGURE 4:**
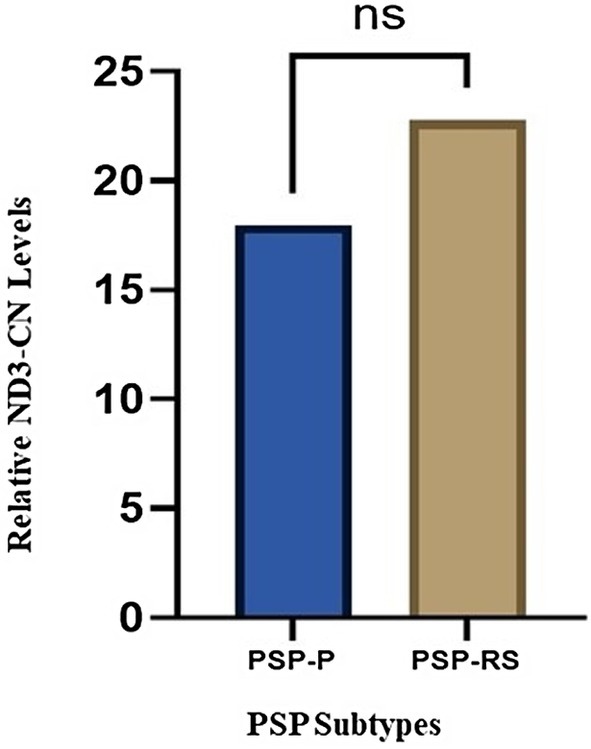
Relative ND3 copy number (CN) levels measured in progressive supranuclear palsy (PSP) patients clustered for disease phenotype.

Our results showed that, although not quite statistically significant, mtDNA‐CN levels were lower in PSP‐P patients compared to PSP‐RS patients (*p* = 0.34) (Figure 4).

**FIGURE 5:**
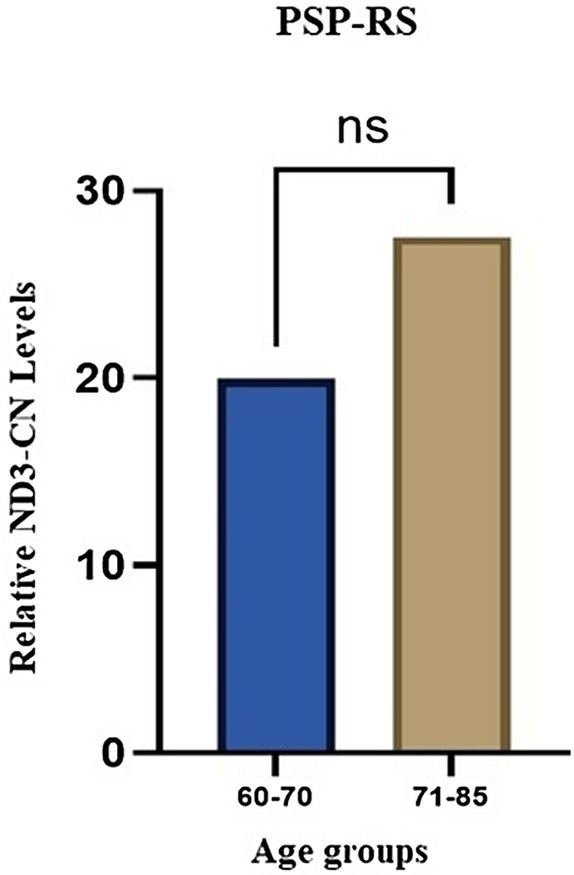
Relative ND3 copy number (CN) levels measured in progressive supranuclear palsy (PSP)‐RS patients clustered for age.

Grouping PSP‐RS patients in two different age ranges, we observed that higher mtDNA‐CN levels were consistently associated with increasing ages, although these data were not statistically significant (*p* = 0.263) (Figure 5).

## 4 Discussion

Our study suggests that alterations in mitochondrial DNA copy number (mtDNA‐CN) may play a meaningful role in the pathoetiology of PSP. mtDNA‐CN represents a promising biomarker to assess mitochondrial health across various human tissues, including peripheral blood, and may hold prognostic value in both the onset and progression of PSP.

Mitochondrial dysfunction and oxidative stress are widely recognized as hallmarks of neurodegenerative diseases [21]. Unlike nuclear DNA, mtDNA lacks protective histones and robust DNA repair mechanisms, making it highly susceptible to oxidative damage from free radicals generated by the electron transport chain (ETC). Impaired mitochondrial function leads to ATP depletion, overproduction of reactive oxygen species (ROS), and subsequent oxidative damage to cellular components, ultimately contributing to neuronal degeneration [22].

Although the precise mechanism linking mitochondrial dysfunction to PSP remains incompletely understood, previous studies have reported reduced activity of the α‐ketoglutarate dehydrogenase complex (KGDHC) in PSP postmortem brain tissues, indicating a potential link between metabolic impairment and disease pathology [23]. Furthermore, cybrid cell lines harboring mtDNA from PSP patients exhibited significantly decreased complex I activity and compensatory increases in antioxidant enzyme activity, indirectly implicating mtDNA mutations in PSP pathogenesis [24].

Our findings show that mtDNA‐CN levels, specifically the *ND3* gene region, are significantly reduced in PSP patients compared to healthy controls (Figure 1). This reduction remained consistent across age groups, suggesting that mitochondrial alterations in PSP may be independent of normal aging or disease progression (Figure 3). These results point toward disrupted mitochondrial biogenesis, defective mitophagy, or impaired oxidative phosphorylation (OXPHOS) efficiency in PSP.

The observed depletion of ND3, a subunit of mitochondrial respiratory complex I embedded in the inner mitochondrial membrane, may be mechanistically linked to tau aggregation. Mitochondrial dysfunction in complex I has been shown to induce oxidative stress and activate tau kinases, leading to hyperphosphorylation and aggregation of tau protein [19]. Tau aggregation may, in turn, amplify neuroinflammatory responses by promoting microglial activation and neurotoxic cascades [25].

We propose that early mitochondrial dysregulation may exacerbate ATP depletion and alter mitochondrial gene expression, driving chronic neuroinflammation and accelerating disease progression. Interestingly, although not statistically significant, our data suggest differences in mtDNA content between PSP clinical phenotypes. PSP‐P patients exhibited lower ND3‐CN levels than PSP‐RS patients (Figure 4), potentially reflecting subtype‐specific mitochondrial vulnerability.

This lower mtDNA‐CN in PSP‐P may be linked to the specific susceptibility of the nigrostriatal pathway to mitochondrial complex I deficits, which is more characteristic of the parkinsonian presentation [26]. In contrast, PSP‐RS is characterized by a more aggressive and widespread tau distribution [5, 27]. The relative “preservation” or slight increase of mtDNA‐CN in some PSP‐RS cases compared to PSP‐P might reflect a compensatory, albeit insufficient, upregulation of mitochondrial biogenesis—a process known as mitohormesis—triggered by higher levels of oxidative stress and chronic neuroinflammation [20, 28].

Furthermore, the clinical features of PSP may be differentially influenced by mitochondrial health. While oculomotor dysfunction and early postural instability in PSP‐RS are driven by rapid tau‐mediated brainstem and cortical atrophy, the predominant akinesia and tremor in PSP‐P might be more closely tied to systemic bioenergetic exhaustion and dopaminergic dysfunction [6]. Mitochondrial dysfunction could thus act as a “threshold” factor; when mtDNA depletion reaches a critical point in the basal ganglia, the parkinsonian phenotype (PSP‐P) dominates [23].

Regarding disease progression, it is observed that many patients initially diagnosed with PSP‐P eventually evolve into the more severe PSP‐RS phenotype [29]. We hypothesize that this clinical transition corresponds to a breakdown in mitochondrial maintenance. In early‐stage PSP‐P, the body may attempt to sequester or limit mitochondrial damage, resulting in a slower progression. However, as “mitoinflammation” becomes chronic, the transition to PSP‐RS may occur when these compensatory mechanisms fail, leading to accelerated tau spreading and the onset of vertical gaze palsy and severe gait instability [30].

The subtle differences in ND3‐CN content between subtypes observed in our study suggest that mitochondrial dynamics may influence the regional deposition of tau [28, 31]. In particular, the higher total and subcortical tau burden typical of PSP‐RS could be driven by greater microglial oxidative stress, potentially linked to localized mtDNA damage [26, 32, 33].

This is further supported by our observation that ND3‐CN levels appeared to increase with age specifically in PSP‐RS patients (Figure 5), which might reflect a reactive, compensatory mitochondrial response to chronic neuroinflammation, underscoring a dynamic interplay between mitochondrial activity and tau pathology throughout the disease course.

## 5 Conclusions

This study highlights the potential role of mtDNA‐CN as a molecular marker of mitochondrial dysfunction and neuroinflammation in PSP. The ability to detect these alterations in peripheral blood offers a less invasive alternative to cerebrospinal fluid analysis and supports the utility of mtDNA‐CN as a biomarker to enhance our understanding of PSP pathophysiology. The main limitation of our work lies in the small sample size, which reflects the rarity of PSP and constrains our ability to thoroughly assess whether mtDNA‐CN alterations vary across PSP phenotypes. Therefore, future investigations with larger patient cohorts and extended follow‐up are essential to confirm our findings and to determine the prognostic relevance of mtDNA‐CN in PSP. Nevertheless, our preliminary evidence underscores the clinical value of monitoring mtDNA‐CN in PSP patients. Peripheral changes in mitochondrial content may reflect the extent of tau pathology and neuroinflammation, thereby aiding in the differential diagnosis and disease monitoring of this challenging neurodegenerative disorder.

## Author Contributions

Luigi Citrigno: conceptualization, methodology, formal analysis, writing – original draft, writing – review and editing. Annamaria Cerantonio: formal analysis, writing – original draft, writing – review and editing. Monica Gagliardi: data curation, writing – review and editing. Radha Procopio: data curation, writing – review and editing. Alessia Felicetti: data curation, writing – review and editing. Anna Aureli: data curation, writing – review and editing. Maurizio Morelli: data curation, writing – review and editing. Grazia Annesi: conceptualization, supervision. All authors read and approved the final manuscript.

## Funding

The authors have nothing to report.

## Ethics Statement

This study adhered to the Declaration of Helsinki, the Council for International Organizations of Medical Sciences International Ethical Guidelines, applicable International Council for Harmonization Good Clinical Practice guidelines, and all applicable laws and regulations of participating countries.

## Conflicts of Interest

The authors declare no conflicts of interest.

## Data Availability

Data used to support the findings of this study are not publicly available to protect participant confidentiality, but they can be available from the corresponding author upon request.
